# Intra-radicular reinforcement using a modified fiber post customization technique: a case report

**DOI:** 10.11604/pamj.2021.40.241.27161

**Published:** 2021-12-20

**Authors:** Usman Anwer Bhatti, Muhammad Qasim Javed, Mustafa Hussein Al Attas

**Affiliations:** 1 Department of Operative Dentistry, Islamabad Medical and Dental College, Islamabad, Pakistan,; 2 Department of Conservative Dental Sciences and Endodontics, College of Dentistry, Qassim University, Buraydah, Saudi Arabia

**Keywords:** Adhesive cement, esthetics, fiberglass, post and core technique, case report

## Abstract

Trauma sustained by developing anterior teeth can lead to an arrested root development and loss of structure which can complicate the endodontic and restorative management. The preservation of the anterior tooth has a definite esthetic and biologic advantage especially during the developmental years of adolescence. However, restorative treatment of such cases is met with serious biomechanical and adhesive challenges in the form of thin dentinal walls, a high configuration factor etc. This case report describes a fiber post customization technique for the intraradicular reinforcement of a maxillary central incisor in a 14-year-old patient.

## Introduction

The prevalence of complicated crown fractures among Pakistani population has been reported to be as high as 27% [1]. The absence of adequate coronal structure associated with such injuries warrants the need for post placement. However, immature teeth with their incomplete root development create an adhesive and biomechanical challenge for conventional post placement. The problem with conventional prefabricated posts in such cases is the inadequate adaptation to the large canal dimensions. Consequently there is accumulation of abundant cement which becomes the vulnerable part of the adhesive interface [2]. Similarly, the placement of a custom fabricated cast post in this situation has the drawback of stress concentration in the apical half of the root making the thin dentinal walls susceptible to fracture.

Intra-radicular reinforcement with fiber post and composite has been reported in literature as a suitable technique for salvaging immature permanent teeth. However, the outcome of this technique is still varying with several factors like age, root conditions, type of reinforcing material and thickness of apical plug affecting it [3-5]. The current case report discusses intra-radicular reinforcement of an immature, structurally compromised anterior tooth in a fourteen years old patient using a technique of fiber post customization for overcoming the adhesive and biomechanical challenges.

## Patient and observation

**Figure 1:**
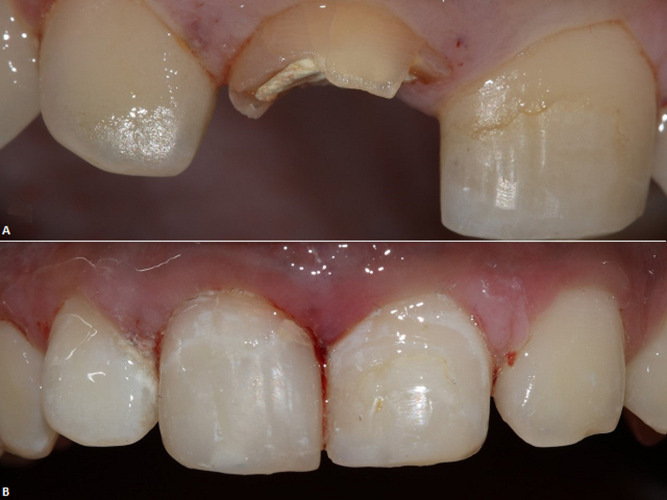
A) preoperative view of the amount of structure loss on maxillary right central incisor; B) postoperative view after completion of the intraradicular reinforcement and composite restoration

**Patient information:** a 14-year-old female patient presented to the department of operative dentistry with a fractured right maxillary central incisor (Figure 1A). The traumatic injury had occurred several years back. The medical history was unremarkable.

**Clinical findings:** a complicated crown fracture of tooth 11 was present with the presence of caries activity on the exposed dentin. The labial mucosa was tender to palpation. A small sinus tract was clinically evident in the attached mucosa.

**Timeline of current episode:** after the initial presentation followed by the formulation of a treatment plan, patient was scheduled for an appointment with the endodontist 2 days later. The endodontist performed the initial debridement and recalled the patient after 1 week. At 1 weeks´ time, canal was re-entered by the same endodontist who performed the prescribed apexification procedure. Finally, the restoration of the tooth was completed 24 hours after the apexification procedure.

**Diagnostic assessment:** percussion test was positive for tooth 11 (FDI). Sinus was traced using a 25 size gutta percha point to the apex of tooth 11 (FDI). The radiographic evaluation revealed periapical radiolucency around immature apex of tooth 11. The mobility and probing depths were within normal limits.

**Diagnosis:** a diagnosis of pulp necrosis and chronic apical abscess secondary to a complicated crown fracture was made. The endodontic prognosis was questionable to fair due to the established lesion and draining sinus tract. The long-term restorative prognosis was poor due to the amount of missing tooth structure.

**Figure 2:**
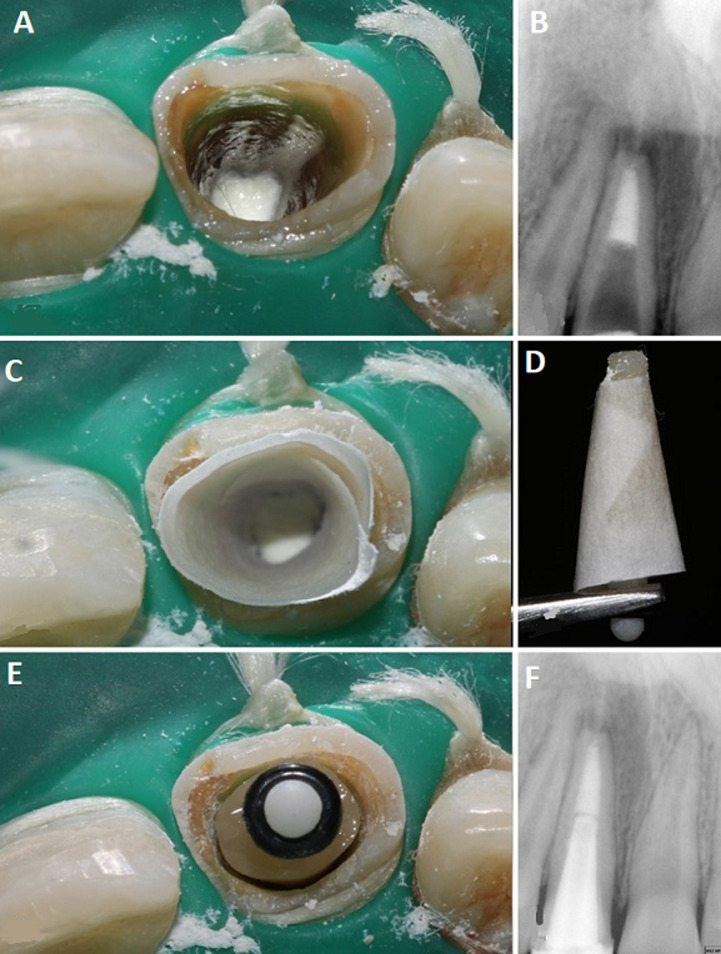
A) placement of MTA as apical barrier; B) radiographic confirmation afer MTA placement, note the thin dentinal walls and large root canal space; C) adaptation of the cellulose paper inside the root canal space to serve as a barrier for prevention of composite entrapment and to allow easy withdrawal of the customized fiber post; D) customized fiber post after removing from the root canal space and before peeling of the cellulose paper; E) checking the fit and adaptation of the customized fiber post; F) postoperative radiograph after completion of intraradicular reinforcement

**Therapeutic interventions:** after discussing different treatment options with the patient and explanation of the guarded prognosis of the treatment it was decided to perform apexification followed by intra-radicular reinforcement and free hand composite build up (Figure 1B) for tooth 11. Isolation of the broken tooth was achieved using a rubber dam clamp positioned distally on the premolar and a floss ligature retained the sheet (Safe Touch, Medicom) over the central incisor. Following completion of the apexification procedure using Mineral Trioxide Aggregate (MTA CEM, NEXOBIO CO, Korea) (Figure 2A, Figure 2B) a period of 24 hours was allowed to lapse for maturation of the MTA before beginning the restoration.

The MTA was inspected for complete setting and the walls of the root canal space were cleaned of any physical remnants of MTA using a microbrush and 17% ethylene diamine tetra-acetic acid (canal plus-septodont). A brief rinse of normal saline followed by rinse of 1% sodium hypochlorite was performed to remove any residual ethylenediaminetetraacetic acid (EDTA) and organic debris. A 1.5 mm diameter glass fiber post (parapost fiber white-choltene whaledent) was treated using a 40% hydrogen peroxide (opalescence-ultradent) for 1 minute. A small piece of cellulose paper was inserted and adapted to the walls of the root canal space (Figure 2C). The post was then covered with a small amount of uncured restorative composite (Te Econom Plus- Ivoclar Vivadent) and positioned inside the root canal space. This was followed by application of additional composite material to allow the adaptation of the composite to the canal circumference before light curing for 20 seconds. After the preliminary curing, the customized fiber post was removed from the root canal space (Figure 2D) and the paper barrier was peeled off to allow additional curing for 20 seconds of the apical half outside the canal, in close proximity to the light source. The customized fiber post was then reinserted to check for adaptation (Figure 2E).

In preparation for adhesive cementation, the intraradicular dentin was treated for 15 seconds using a 37% phosphoric acid (Eco Etch-Ivoclar Vivadent) followed by 15 seconds rinsing with water and drying with air and paper points. For rewetting the dentin, 0.12% chlorhexidine solution (Clinica) was applied for 30 seconds and then excess solution was drained using cotton pellet and paper points. An adhesive system comprising of primer and adhesive resin (Adper single bond2- 3M) was applied using a microbrush by actively scrubbing the dentin walls for 20 seconds followed by air drying for 10-15 seconds to volatilize the solvent. The adhesive was light cured for 1 minute in the presence of a large fiber post to allow distribution of light to the depth of the root canal space. Dual cure adhesive resin cement was generously introduced into the root canal space using a lentulospiral (size 40-Mani). The customized fiber post was inserted, and excess cement wiped using a microbrush following which light curing was carried out for 60 seconds. A direct composite restoration (Te Econom Plus-Ivovlar Vivadent) was completed over the customized fiber post and a radiograph was taken for final assessment (Figure 1B, Figure 2F).

**Follow-up and outcome of interventions:** an intact restoration with absence of any signs and symptoms of periapical disease was observed at the 6- and 12-months clinical follow-up.

**Patient perspective:** at 12 months follow up patient was satisfied with the aesthetic result and the resolution of sinus tract.

**Informed consent:** an informed consent was taken from the patient and her primary care giver (mother).

## Discussion

During intra-radicular reinforcement, the presence of an intermediate layer of composite between the post and the dentinal walls has been found to improve the fracture resistance of immature teeth [6]. This case report demonstrates a predictable way to adapt composite in the intra-radicular area while attempting to overcome the adhesive challenges associated with intra-radicular reinforcement.

The conventional technique for intra-radicular reinforcement relies on a light transmitting fiber post which can allow better light distribution to the root canal depth for curing of the light cured composite material [2]. Although the light cured composite offers adequate working time for adjusting the post position, the degree of polymerization remains questionable. Alternatively the use of a chemically cured composite material lacks the advantage of extended working time needed for correct post alignment [2]. Moreover, chemically cured materials have issues of incompatibility with simplified adhesives and a relatively limited degree of polymerization compared with light cured materials [7]. Since simplified adhesives are popular among majority of the dental practitioners due to the benefits of low cost and ease of use, it becomes relevant to devise techniques which are compatible with such adhesives.

The major adhesive challenges of intraradicular reinforcement include the high configuration-factor (C-factor) [7,8], presence of an altered bonding substrate, presence of sealer/gutta percha infiltrated smear layer [9], suboptimal polymerization of the reinforcing composite [10] and cement voids [11,12]. While exclusively relying on MTA for the apical seal and avoiding gutta percha/sealer we can overcome the adhesive challenge posed by their presence. But the challenges posed by high C-factor and suboptimal polymerization of reinforcing composite continue to threaten the achievable bond with the radicular dentin.

In the technique described by the authors the reinforcing composite was adapted and cured prior to adhesive bond development with radicular dentin thus preventing the detrimental effects of curing composite bulk in a high C-factor situation. Additionally, after achieving complete polymerization of the reinforcing composite a customized fiber post is produced which has a close adaptation to the radicular dentin minimizes the volume of resin cement required for adhesive cementation. The reduced volume of resin cement exhibits reduced polymerization shrinkage with a reduced likelihood of gap development [12]. These effects would have been more pronounced had bulk curing of the composite was achieved with simultaneous bond development with radicular dentin using a light transmitting post.

## Conclusion

The sequential development of an adhesive bond in the order described in this case report, that is, between: 1) Reinforcing composite; 2) fiber post and; 3) radicular dentin in contrast to simultaneous bond development as previously described in literature simplifies clinical procedure with the potential improvement of adhesion in intra-radicular reinforcement.
